# Population structure of the ash dieback pathogen, *Hymenoscyphus fraxineus*, in relation to its mode of arrival in the UK

**DOI:** 10.1111/ppa.12762

**Published:** 2017-09-26

**Authors:** E. S. Orton, C. M. Brasier, L. J. Bilham, A. Bansal, J. F. Webber, J .K. M. Brown

**Affiliations:** ^1^ John Innes Centre Norwich Research Park Norwich NR4 7UH UK; ^2^ Forest Research Alice Holt Lodge Farnham GU10 4LH UK; ^3^ Present address: Plant Impact plc Rothamsted, West Common Harpenden AL5 2JQ UK

**Keywords:** *Hymenoscyphus fraxineus*, mating type, population structure, route of pathogen transmission, vegetative compatibility

## Abstract

The ash dieback fungus, *Hymenoscyphus fraxineus*, a destructive, alien pathogen of common ash (*Fraxinus excelsior*), has spread across Europe over the past 25 years and was first observed in the UK in 2012. To investigate the relationship of the pathogen's population structure to its mode of arrival, isolates were obtained from locations in England and Wales, either where established natural populations of ash had been infected by wind‐dispersed ascospores or where the fungus had been introduced on imported planting stock. Population structure was determined by tests for vegetative compatibility (VC), mating type and single‐nucleotide polymorphisms (SNPs). VC heterogeneity was high at all locations, with 96% of isolate pairings being incompatible. Frequencies of the *MAT1‐1‐1* and *MAT1‐2‐1* idiomorphs were approximately equal, consistent with *H. fraxineus* being an obligate outbreeder. Most SNP variation occurred within study location and there was little genetic differentiation between the two types of location in the UK, or between pathogen populations in the UK and continental Europe. There was modest differentiation between UK subpopulations, consistent with genetic variation between source populations in continental Europe. However, there was no evidence of strong founder effects, indicating that numerous individuals of *H. fraxineus* initiated infection at each location, regardless of the route of pathogen transmission. The ssRNA virus HfMV1 was present at moderate to high frequencies in all UK subpopulations. The results imply that management of an introduced plant pathogen requires action against its spread at the continental level involving coordinated efforts by European countries.

## Introduction

Increasing global trade has resulted in the dispersal of numerous pests and pathogens across the world on imported plants, including many tree pathogens (Brasier, 2008; Roy *et al*., 2014). The role of trade in these introductions is illustrated by the positive correlation between high trade volumes and the number of introduced pests and pathogens in many countries around the world (Roy *et al*., 2014). Many of these introductions have led to devastating disease outbreaks, especially when a parasite has encountered a highly susceptible host (Brasier, 2008). Destructive pathogens of trees that have recently been introduced by trade into the UK include *Phytophthora ramorum*,* P. alni*,* P. lateralis*,* P. austrocedri*,* Cryphonectria parasitica* and *Dothistroma septosporum* (Freer‐Smith & Webber, 2015).

Natural processes, notably wind‐dispersal, have also been implicated in the spread of some parasites from sites of introduction to new areas. This has often been particularly important in expanding the range of pathogens in agriculture, especially when a crop has been introduced to a new area. In several instances, major epidemic diseases that have been introduced to new locations by human activity have then been spread rapidly by natural dispersal. Examples include the spread of potato late blight (*P. infestans*) across Europe in the early nineteenth century and black leaf streak of banana (*Mycosphaerella fijiensis*) throughout the tropics in the second half of the twentieth century (Brown & Hovmøller, 2002).

A destructive epidemic dieback of *Fraxinus excelsior* (common European ash), caused by the ascomycete fungus *Hymenoscyphus fraxineus* (syn. *Chalara fraxinea)*, is currently occurring across much of Europe. Symptoms were first observed in Poland in the early 1990s, but, initially, were not linked to a specific pathogen (Gross *et al*., 2014a). By the time the causal agent had been identified (Gross *et al*., 2014a), the pathogen had spread rapidly and widely, causing heavy mortality of ash trees in many European countries and replacing the endemic European ash foliage colonizer *H. albidus* (Pautasso *et al*., 2013). It was first detected in the UK and in Ireland in 2012 (http://www.agriculture.gov.ie/press/pressreleases/2012/October/title,67002,en.html; Freer‐Smith & Webber, 2015).

*Hymenoscyphus fraxineus* is thought to be native to East Asia. It has been identified from indigenous ash species in Japan (Zhao *et al*., 2012), Korea (Han *et al*., 2014), China (Zheng & Zhuang, 2014) and the far east of the Russian Federation (Drenkhan *et al*., 2017) but the source of the genotypes causing the outbreak in Europe is unknown. However, it is clear that East Asian populations are strongly differentiated from those in Europe and have much greater genetic diversity, as shown by simple sequence repeat (SSR; microsatellite) markers (Zhao *et al*., 2012; Gross *et al*., 2014a; Cleary *et al*., 2016), rDNA ITS sequences (Drenkhan *et al*., 2017) and genome sequencing (Yoshida *et al*., 2013).

*Hymenoscyphus fraxineus* is considered to be an obligately sexual outcrossing fungus (Gross *et al*., 2014a). In summer, ascocarps develop profusely from pseudosclerotia on fallen rachises produced from leaves infected during the previous year. These fruiting bodies release wind‐dispersed ascospores that are the primary means of dispersal of the pathogen, with recorded levels of inoculum reaching 100 spores m^−3^ (Chandelier *et al*., 2014). Spores infect leaves, including rachises, and from here the fungus can spread to initiate lesions on branches and stems, killing the cambium and causing vascular dysfunction so that distal host tissue then wilts and dies.

It is probable that both windborne spores from Europe and imports of already infected ash saplings from Europe were involved in the introduction of *H. fraxineus* into the UK. The first confirmed identifications of the pathogen in the UK were at a nursery followed by a recent landscape planting in March and May 2012, respectively (Freer‐Smith & Webber, 2015). The UK has been a substantial importer of trees from continental Europe. Between 2003 and 2011, five million ash saplings were imported (Sansford, 2013) even though it was known, at least during the latter half of that period, that *H. fraxineus* was causing widespread damage to ash in exporting countries (Pautasso *et al*., 2013). The distribution of early findings of ash dieback in the UK and Ireland suggested that while windborne dispersal of ascospores from continental Europe caused infection of woodlands near the east coast of England and Scotland, *H. fraxineus* was also introduced on imported trees planted at many other locations (Freer‐Smith & Webber, 2015). In autumn 2012 advanced dieback symptoms were confirmed in ash trees at several locations in East Anglia where there had been no recent planting, including the first site where the disease was detected in the wider environment in South Norfolk District (http://www.wildlifetrusts.org/news/2012/10/25/ash-dieback-disease-found-norfolk; location LWD‐E in this paper). Therefore, it is likely that multiple airborne incursions of the pathogen infected woods in eastern Great Britain.

Previous studies of genetic diversity of *H. fraxineus* in Europe using SSR markers have indicated that European isolates come from the same source population and that differentiation between subpopulations is small, allelic variation is low and genotypic diversity is high (Gross *et al*., 2014a; Burokiene *et al*., 2015; Haňáčková *et al*., 2015). The only study of phenotypic variation to date reported diversity in vegetative compatibility (VC) types, a system that defines genetic individuals within a fungal population (Brasier & Webber, 2013). Vegetative compatibility in ascomycete fungi is controlled by a system of multiple genes, in which there is compatibility between two interacting fungal individuals if they both have the same alleles at all loci. Vegetatively incompatible pairings of *H. fraxineus* isolates produce a distinctive ‘gap’ reaction and a high diversity of VC types has been found at some UK locations (Brasier & Webber, 2013). In other fungi, diversity in VC types is correlated with genetic diversity, e.g. *Cryphonectria parasitica*, the chestnut blight pathogen (Liu *et al*., 1996). In the Dutch elm disease pathogen, *Ophiostoma novo‐ulmi*, it probably drives outcrossing (Brasier, 1986).

This paper reports a study of population genetics of *H. fraxineus* at diverse locations in the UK. From intensive disease surveys carried out after the initial discovery of ash dieback in the UK, it was possible to classify disease locations according to the probable source of infection. Ash dieback at many established woods near the east coast was probably caused by windborne spores arriving from continental Europe, because these outbreaks occurred independently of records of recent plantings of imported ash. The appearance of ash dieback symptoms at isolated early outbreak locations further west, however, was closely associated with plantings of imported, potentially infected nursery stock. The question explored in this study was whether the route of entry influenced the pathogen's genetic diversity and population structure at disease outbreak locations across the UK. In a structured survey of *H. fraxineus* subpopulations at three established sites and three planted locations, variation in VC types, mating types and single‐nucleotide polymorphism (SNP) markers was studied, as well as the frequency of an ssRNA virus, Hymenoscyphus fraxineus mitovirus 1 (HfMV1), already identified in European *H. fraxineus* populations (Schoebel *et al*., 2014, 2017). The hypothesis tested was that the amount of genetic variation at the two different types of outbreak location was related to the mode of arrival in the UK, with the level of differentiation between subpopulations reflecting the size and diversity of the founding populations. The comparative genetic diversity at these two types of site provides insight into the processes of ash infection and the behaviour of the ash dieback pathogen in the UK.

## Materials and methods

### Locations, sample collection and fungal isolation

**Figure 1:**
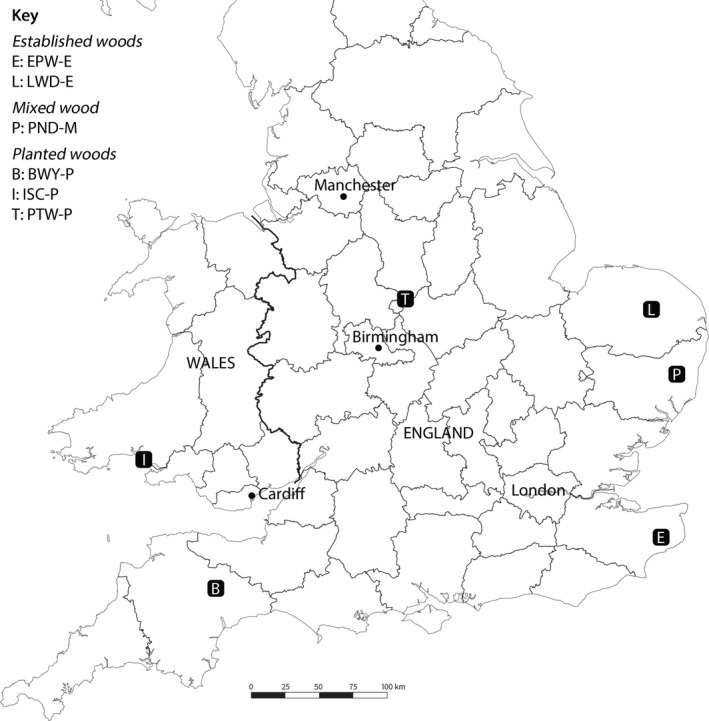
Map showing the locations of the populations sampled in England and Wales in 2013.

Infected sections of *F. excelsior* stems with visible bark lesions, were collected from six locations between December 2013 and May 2014 (Fig. 1; Table S1). The disease was therefore in its epidemic phase at each location. At the time of sampling, ash dieback was widespread in natural woodland and the wider environment throughout southeast England and occurred frequently in recently planted trees throughout the UK (https://www.forestry.gov.uk/forestry/infd-8udm6s). LWD‐E and EPW‐E are established, seminatural ancient woodlands in eastern England with no record of recent planting, so infection was presumed to have been initiated by airborne spores from continental Europe. At the time of sampling, there was also prolific infection in the wider natural environment at these locations. ISC‐P, BWY‐P and PTW‐P were isolated, highly localized but heavily infected disease locations in central or western England and south Wales where ash saplings had been planted over the previous 10–20 years. As no infection was observed in nearby ash trees outside the planted area, *H. fraxineus* was presumed to have arrived on infected planting stock. PND‐M is in eastern England and has both established ash trees and trees planted between 1993 and 2002. While it may have been infected by both routes, it is located well within the area of widespread natural infection.

Stems were selected for sampling if they had at least one lesion or lesions that appeared to have been formed in recent months and had not yet cankered. Stems were wiped with 70% ethanol and dieback lesions exposed by paring away the outer bark to expose necrosis in the underlying phloem. Small pieces at the edge of the visible lesion were then cut out using a sterile scalpel and placed on 2% malt agar (MA) supplemented with 0.02% streptomycin (MA+S) (Brasier & Webber, 2013). Plates were incubated at 20 °C in the dark. Any fungal growth that appeared to be *H. fraxineus* was quickly excised and subcultured onto fresh MA + S plates. Only one isolate from each lesion was retained for study. Cultures could not be obtained from all lesions sampled because the fungus can be difficult to grow in culture.

### Vegetative compatibility tests

Tests for vegetative compatibility or incompatibility between pairs of isolates were carried out as described by Brasier & Webber (2013). Isolates were paired on both ash sapwood agar (ASA) and MA. ASA was modified to contain only 30 g L^−1^ of milled ash sapwood. Self–self pairings (the same isolate inoculated twice on a plate) of each isolate were set up in duplicate as compatible controls. Pairings were repeated if an isolate failed to grow or had sectored making it difficult to score, or if the plate was contaminated. Pairings resulting in a clear gap reaction were not repeated. Several isolates, including all those from PTW‐P, were unstable on MA, so tests involving these isolates were repeated on ash leaf malt agar (ALMA; Gross *et al*., 2012 amended to 30 g of ash leaf tissue per litre), which reduced sectoring. Results were consistent if isolate pairings grew on both MA and ALMA without sectoring. After a minimum of 4 weeks' incubation at 20 °C in darkness, all pairings were scored as compatible (colonies intermingling) or incompatible (a structured gap reaction between the colonies) according to the terminology of Brasier & Webber (2013), but no assessment was made for the incompatible‐associated L‐reactions.

### DNA extraction

Isolates were grown by putting a mycelial plug from culture plates in 50 mL of malt extract broth (20 g L^−1^) and incubating at 20 °C in the dark for 2–4 weeks. Resultant mycelia were freeze dried and genomic DNA was extracted using a modified CTAB method (Kohler *et al*., 2011) without the purification step.

### Species validation and mating type analysis

Species validation and determination of mating‐type of the isolates was carried out using a PCR method (Gross *et al*., 2012). Reactions for mating type idiomorphs *MAT1‐1‐3* and *MAT1‐2‐1* were carried out separately with the modification that annealing temperatures were changed to 60 and 55 °C, respectively, to reduce the appearance of nonspecific bands in the PCR assay for *MAT1‐1‐3*. Deviations of *MAT1* allele frequencies from a 1:1 ratio at each location were tested by a χ^2^ test.

### Sequence analysis

Identification of SNP markers was based on a comparison between Version 1 of the genome sequence assembly of *H. fraxineus* isolate KW1 from Kenninghall Wood, Norfolk (https://geefu.oadb.tsl.ac.uk/) and an additional 42 isolates of *H. fraxineus* from the UK, France, Norway and Poland, sequenced by the Earlham Institute, Norwich and Edinburgh Genomics (www.geefu.oadb.tsl.ac.uk). The aligned sequence scaffolds of all 43 isolates were examined manually for SNPs and variable sites were selected if there was a polymorphism in both UK and continental isolates with a frequency of 24–78% overall. SNPs from both coding and noncoding regions were included but variants within 5 kb of the end of each scaffold were avoided. When Version 2 of the genome assembly became available, a blast analysis of the selected SNP regions confirmed that SNP markers were distributed uniformly throughout the genome.

### Primer design and SNP analysis

Primers for SNP detection by Kompetitive Allele Specific PCR (KASP; LGC Genomics) were designed using polymarker (Ramirez‐Gonzalez *et al*., 2015). For each locus, a common primer and two allele‐specific primers were used with either a 5′‐HEX‐GAAGGTCGGAGTCAACGGATT‐3′ or 5′‐FAM‐GAAGGTGACCAAGTTCATGCT‐3′ adapter tail, each primer being specific to one of the two alleles. All primers and the location of each SNP are listed in Table S2. Primer sets were tested initially on a minimum of 23 individuals from two populations to ensure that there was clear allele separation, leading to the selection of 28 markers. Genotyping was performed according to the manufacturer's instructions using a Mastercycler Pro 384 well thermocycler (Eppendorf). Primers were validated against DNA from selected previously sequenced isolates provided by the Food and Environment Research Agency (Fera; http://oadb.tsl.ac.uk/). For each assay at least one no‐template‐control (NTC) was included. Cycling conditions were 94 °C for 15 mins; then 10 cycles of 94 °C 20 s, 65–57 °C 1 min (decreasing 0.8 °C per cycle); and finally 36 cycles of 94 °C 20 s, 57 °C 1 min. Fluorescence was measured using a Safire (XFluor 4) plate reader (Tecan) and alleles were identified using klustercaller software (LGC).

### Analysis of SNP data

SNP data was analysed using the poppr package (Kamvar *et al*., 2014) in the R statistical programming language. For each subpopulation, the number of multilocus genotypes, the Shannon–Wiener index, Nei's gene diversity (Nei, 1973) and the standardized index of linkage disequilibrium *r̅*_d_ were calculated (Agapow & Burt, 2001). The poppr package was also used to calculate AMOVA. Genetic differentiation (*G*_ST_) between pairs of populations was calculated from data on all 28 SNP loci by the method of Nei (1973). The statistical significance of *G*_ST_ was calculated by a randomization procedure in which all isolates in the respective populations were reassigned randomly to the populations and *G*_ST_ recalculated from the randomized dataset. This simulated the distribution of *G*_ST_ under the null hypothesis of no differentiation between populations, enabling the construction of significance tests. GenStat 18th edition (VSN International) was used to do a principal coordinates analysis of the SNP data for the six UK subpopulations and for these UK isolates plus the continental European isolates used for SNP discovery.

### Presence of HfMV1

Superscript III (Invitrogen) was used to synthesize cDNA from RNA extracted from isolates collected from all six subpopulations and this was analysed for the presence of ssRNA virus HfMV1. The method of Schoebel *et al*. (2014) was followed except that the PCR for the actin control and the ssRNA were run separately, not as a multiplex.

## Results

### Vegetative compatibility (VC) tests

Within each location, isolates were tested for VC in all possible pairing combinations (Fig. S1). Compatible reactions between isolates, except self–self pairings, were rare in all six subpopulations (Table 1). In most pairings there was a clear, uniformly structured, 1–3‐mm‐wide gap reaction between the colonies, demonstrating an incompatible phenotype. Some reactions were difficult to score because of heavy sectoring, because one isolate grew much faster than the other, or because only a very narrow (<1 mm) gap formed between the isolates. A few pairings produced unreadable reactions because of heavy sectoring even after repeat tests (Fig. S2) and were excluded from the analysis. Moreover, not all self–self duplicate pairings developed in an identical way, as sometimes one plate exhibited full colony intermingling and the other exhibited a diffuse, unstructured gap at the margins of the colony junction line. This indicates the potential for minor variability in VC tests (Brasier & Webber, 2013; Brasier *et al*., 2017). Tests on ASA and either MA or ALMA gave consistent results for most pairs of isolates with different scores being recorded in only a few cases (BWY‐P 007+010; EPW‐E 082+076; PND‐M 007+016; PTW‐P 039+041d and PTW‐P 031+048).

**Table 1:** Comparison of vegetative compatibility (VC) tests on isolates of *Hymenoscyphus fraxineus* collected at three established (−E) or mixed (−M) woodland sites and three planted (−P) sites

Subpopulation	Agar	No. of isolates	Scored pairs	Compatible pairs		VC types (min.–max.)	χ^2^
				No.	%		
BWY‐P	MA/ALMA	8	28	2	7	6	0.02
	ASA	8	28	1	4	7	0.36
ISC‐P	MA/ALMA	13	74	4	5	9–10	0.12
	ASA	13	71	5	7	9–10	0.04
PTW‐P	ALMA	10	45	8	18	7–9	9.07
	ASA	10	44	8	18	7–9	9.38
EPW‐E	MA/ALMA	13	77	4	5	10–11	0.18
	ASA	13	77	5	6	9–11	0.00
LWD‐E	MA/ALMA	10	43	1	2	9	1.12
	ASA	10	45	1	2	9	1.24
PND‐M	MA/ALMA	10	45	1	2	10	1.23
	ASA	10	45	0	0	10	2.90
Total	MA/ALMA						11.70^a^
	ASA						13.90^a^

Isolate pairings were grown on malt agar (MA) and/or ash leaf malt agar (ALMA) and ash sapwood agar (ASA) and scored for presence or absence of a gap reaction; absence of the gap reaction indicated a compatible pairing. Chi‐squared calculated for compatible pairings on each type of agar with 5 d.f.

a Indicates significant at the 5% confidence level.

There was high local VC diversity at all six locations, with 93.8% of pairings on ASA and 93.3% on MA or ALMA recorded as incompatible reactions. Subpopulations from LWD‐E and PND‐M had the lowest frequencies of compatible reactions, indicating the highest diversity of VC types, estimated by calculating the minimum and maximum number of different VC genotypes required to account for the observed pattern of interaction (Table 1). The PTW‐P subpopulation had the highest percentage of compatible reactions, but was also the most difficult to score because of heavy sectoring by several isolates on ALMA.

### Genetic diversity and differentiation

A total of 119 SNP markers distributed throughout the genome were identified by comparing genome sequences of *H. fraxineus*, and primers were designed for use in a KASP system (Table S2). All SNP markers were biallelic and no multiallelic loci were found during the selection stage. In this study 28 markers, with one or two from each genome sequence scaffold, were used.

Within each subpopulation, the index of association between all the SNP markers, which estimates multilocus linkage disequilibrium (LD) using the *r̅*_d_ statistic (Agapow & Burt, 2001), ranged from −0.0052 to 0.0523. There was no significant evidence (*P *> 0.05) for LD of genetic loci in any subpopulation except PND‐M (*P *< 0.001; Table 3). When all 90 UK isolates were analysed as a whole, however, there was evidence for significant LD of the loci analysed (*r̅*_d_ = 0.0206; *P *< 0.001).

Nei's gene diversity (expected heterozygosity, *H*_*S*_; Table 2) ranged from 0.294 (PTW‐P) to 0.451 (PND‐M) with an average of 0.411. This indicated high genetic diversity throughout the population because the maximum *H*_*S*_ possible with two alleles per locus in a haploid organism is 0.5. Both mating types were found at all locations and, in the UK as a whole as well as for all six subpopulations, the ratio of mating types did not differ significantly from the 1:1 ratio expected in a regularly outbreeding population (Table 2).

**Table 2:** Genotypic diversity, linkage disequilibrium, frequency of *MAT1‐1* and presence of HfMV1 ssRNA virus for *Hymenoscyphus fraxineus* sampled from three established (−E) or mixed (−M) locations and three planted (−P) locations

Subpopulation^a^	*N*	*H*	No. of monomorphic SNP markers	*H*_S_	*r̅*_d_	*P* (*r̅*_d_)	*MAT1‐1 %* (*P* χ^2^)	HfMV1 % (no. tested)
EPW‐E	19	2.94	3	0.354	−0.0052	0.97	32 (0.3)	67 (15)
LWD‐E	13	2.56	6	0.309	0.0130	0.14	54 (0.8)	20 (10)
PND‐M	14	2.64	0	0.451	0.0523	<0.001	71 (0.3)	100 (13)
BWY‐P	10	2.30	3	0.365	0.0098	0.14	80 (0.2)	46 (13)
ISC‐P	14	2.64	7	0.343	0.0311	0.14	36 (0.5)	87 (15)
PTW‐P	20	3.00	5	0.294	0.0107	0.64	40 (0.5)	67 (24)
Total	90	4.50		0.411	0.0206	<0.001	49 (0.9)	67; *P *= 0.0007

28 SNP markers were tested on each population.

*N*, sample size; *H*, Shannon–Wiener index; *H*_S_, Nei's gene diversity (expected heterozygosity); *r̅*_d_, standardized index of multilocus linkage disequilibrium (Agapow & Burt, 2001); *P*, value for significance of *r̅*_d_. The *MAT1‐1* allele frequencies at each site were tested for deviation from a 1:1 ratio using a χ^2^ test with 1 degree of freedom. Homogeneity of the frequency of HfMV1 across sites was tested by a χ^2^ test (5 d.f.).

a For details of subpopulations see Table S1.

Each of the 90 isolates tested had a unique multilocus genotype with the 28 SNP markers, even though some data points were missing. A total of 59 isolates were scored for the entire set of 28 markers, 13 isolates lacked data for one marker and a further five lacked data for two markers. A further 13 isolates had more than two missing data points, up to a maximum of 13. In several of the PND‐M isolates (002, 003, 004, 006, 007, 008, 009, 010 and 011) SNP markers Hf926:10136 (except PND‐M 009 and 010) and Hf945:18575 could not be amplified even after repeated attempts using KASP. It was hypothesized that a third allele might be present, but sequence analysis of these regions showed one or other of the two expected SNP alleles in all these isolates. The *G*_ST_ value between all subpopulations was 0.20 (*P *< 0.0001), indicating moderate differentiation. In a comparison of the isolates from established or mixed woods in eastern England with those from planted woods located further west, *G*_ST_ was 0.04, indicating very limited differentiation between the two groups. In pairwise comparisons between subpopulations, *G*_ST_ ranged from 0.04 to 0.19 (Table 3). AMOVA confirmed these findings, indicating that 18% of the total variation was distributed between populations (Φ_ST_ = 0.18, *P *= 0.001) and the remainder within populations. Comparing the established or mixed sites with the planted sites, 5% of the total variation was due to differences in the mode of arrival (Φ_ST_ = 0.046, *P *= 0.001).

**Table 3:** Matrix of pairwise population differentiation (*G*_ST_) based on SNP marker data for six subpopulations of UK *Hymenoscyphus fraxineus* at established (−E), mixed (−M) or planted (−P) sites

Subpopulation^a^	EPW‐E	LWD‐E	PND‐M	BWY‐P	ISC‐P	PTW‐P
EPW‐E	0					
LWD‐E	0.19***	0				
PND‐M	0.04 ns	0.13***	0			
BWY‐P	0.13**	0.19***	0.11**	0		
ISC‐P	0.11**	0.15***	0.09**	0.13***	0	
PTW‐P	0.12***	0.20***	0.06 ns	0.15***	0.13***	0

The statistical significance of *G*_ST_ was calculated by a randomization procedure. ns, not significant, **P < 0.1, ***P < 0.001. Planted vs. Established/Mixed sites ***P = 0.04. Between all subpopulations ***P = 0.20.

Details of subpopulations are in Table S1.

Among the 43 isolates used to identify SNPs, the 20 isolates that came from continental Europe were compared to the UK isolates reported here using the 28 selected SNP markers. This revealed very low genetic differentiation between *H. fraxineus* populations of continental Europe and those from the eastern established or mixed locations (*G*_ST_ = 0.05) or the more westerly‐planted locations in the UK (*G*_ST_ = 0.06). Therefore, UK subpopulations were generally less genetically differentiated from the continental European population than they were from each other. Allelic diversity was well distributed over the six UK locations, with all subpopulations polymorphic for most SNP markers (Table 2). No subpopulation had a private allele of any of the SNP markers tested and even the least common allele was present in at least three subpopulations.

**Figure 2:**
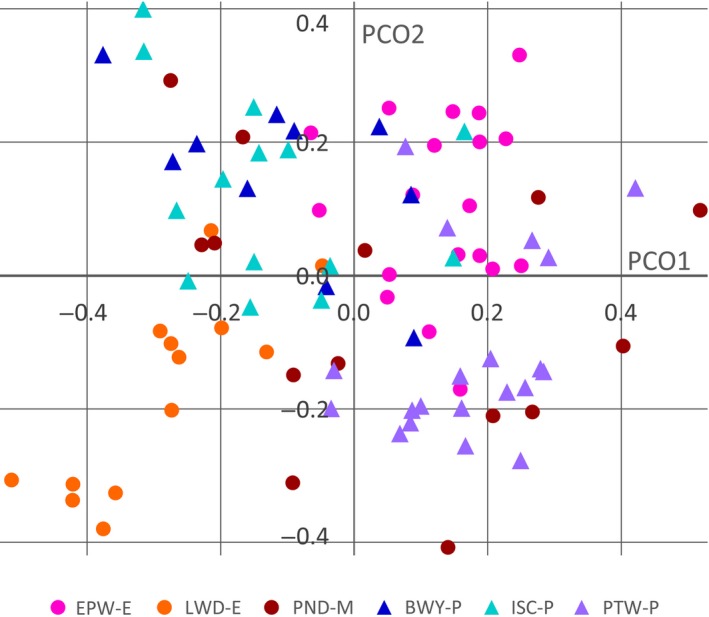
Principal coordinates analysis of single‐nucleotide polymorphisms (SNP) in the six UK subpopulations of *Hymenoscyphus fraxineus*. Isolates from established sites are shown by red, pink and orange circles and isolates from planted sites are shown by blue, purple and turquoise triangles. Subpopulations are not separated from each other on the first two principal coordinates except for EPW‐E and LWD‐E, which are separated from each other but not from any other subpopulation.

In a principal coordinate (PCO) analysis of the SNP data (Fig. 2), the first three PCOs accounted for 11.89%, 8.86% and 8.27% of the total variation (average variation for 28 PCOs = 3.6%). There was minor variation between the six subpopulations, which occupied overlapping but not identical regions of PCO space. This was most striking for LWD‐E and EPW‐E, which overlapped only slightly on the first two PCOs, and for LWD‐E and PTW‐P, which did not overlap on the first PCO. The most diverse population, PND‐M, overlapped with all other subpopulations on the first two PCOs, which was reflected in low *G*_ST_ values for PND‐M compared with each of the other locations. There was very little differentiation between isolates from the easterly established or mixed locations and the planted locations in the west, reflecting the low *G*_ST_ values for these comparisons. In a PCO analysis comparing the UK subpopulations with continental European isolates, the first three PCOs accounted for 8.75%, 6.56% and 6.44% of the total variation. The PCO plot (Fig. S3) showed that the UK isolates were not differentiated from continental European isolates.

### ssRNA virus

HfMV1 was detected in a high proportion of all *H. fraxineus* isolates tested, although there was significant variation in its frequency between the UK subpopulations (χ^2^ test = 21.5, 5 d.f., *P *= 0.0007). Overall, it was present in 67% of isolates but there was an especially low frequency of 20% at LWD‐E and a high frequency of 100% at PND‐M (Table 2; contributions of LWD‐E and PND‐M to the χ^2^ statistic were 9.8 and 6.5 respectively).

## Discussion

The pattern of ash dieback outbreaks indicates that *H. fraxineus* has entered the UK both on imported, infected planting stock and by wind‐dispersed ascospores from disease outbreaks in continental Europe (Sansford, 2013). In the present study, subpopulations of *H. fraxineus* at six UK locations from ash trees infected by either process were compared using VC tests, SNP markers, *MAT1* idiomorph frequencies and the presence of HfMV1.

VC tests demonstrated high levels of heterogeneity, with few isolates in the same subpopulation proving to be compatible with each other. This is consistent with the observations of Brasier & Webber (2013), who also found high levels of incompatibility (91–98%) between isolates from three UK ash dieback locations. The three established or mixed locations infected by windborne inoculum and two planted locations had similar, high diversity in VC types while PTW‐P, a planted location, had a higher proportion of compatible isolate pairings, although the majority were incompatible. The reason for this difference is unknown but may be due to scoring error given the high level of sectoring exhibited by some PTW‐P isolates, as discussed below. There was no evidence from the other indicators of variation to suggest that the population at PTW‐P was less genetically diverse than the other study subpopulations.

SNP marker alleles revealed little genetic differentiation, with low *G*_ST_ values between *H. fraxineus* from continental Europe and the two categories of UK subpopulation, whether from planted or established/mixed locations. All marker alleles present in the UK isolates were also found in the European isolates. Additionally, there was little differentiation between the two categories of location and the great majority of genetic diversity in the UK population of *H. fraxineus* was within subpopulations, not between them.

As would be expected for a random‐mating and routinely outcrossing species, and as previously observed for populations in continental Europe (Gross *et al*., 2012), the frequencies of the two mating types were not significantly different. Nevertheless, the UK population as a whole was in significant linkage disequilibrium, as also was the subpopulation at PND‐M, although the other subpopulations were not. At the UK level, linkage disequilibrium may be an artefact of pooling diverse subpopulations with different allele frequencies (Wolfe & Knott, 1982). This may also have been the case at PND‐M, where the *H. fraxineus* subpopulation may have come from separate sources on the continent: from airborne ascospores infecting established trees and from local inoculum coming from the source nurseries of the planted trees.

In continental Europe, the prevalence of the ssRNA virus HfMV1 was highly variable between different countries, ranging from 0% in Belgium and Slovenia to 91% in Switzerland (Schoebel *et al*., 2017). Comparable virus frequencies were found at the different locations in the UK, ranging from 20% at LWD‐E to 100% at PND‐M. As HfMV1 has no known detrimental impact on the fungus (Schoebel *et al*., 2014), its frequency may be influenced by stochastic population dynamics. It has been suggested that VC may act as a barrier to the spread of cytoplasmic elements such as viruses (Brasier & Webber, 2013). The high heterogeneity in VC types implies that even if a deleterious virus were present, it could not spread rapidly and widely between fungal mycelia within a subpopulation. The high but variable frequency of HfMV1 is in accordance with the virus being transmitted and spread mainly via the ascospores (Schoebel *et al*., 2017).

Collectively, the present observations on VC, SNP markers, mating types and virus frequency point to the UK population being founded by numerous genetic individuals from continental Europe, although *H. fraxineus* throughout Europe appears to have originated from a single founder population (Gross *et al*., 2014b; Burokiene *et al*., 2015; Schoebel *et al*., 2017) followed by long‐distance dispersal by means of sexually produced spores. The results of the present study, demonstrating many unique individuals at each location, do not provide evidence for clonal reproduction and consequently do not indicate a role for substantial dispersal by asexual inoculum as proposed by Fones *et al*. (2016). The limited structure within the UK and European populations is consistent with that previously observed in Europe using SSR markers (Gross *et al*., 2012, 2014a; Haňáčková *et al*., 2015; Nguyen *et al*., 2016) and supports the hypothesis that the *H. fraxineus* population has spread recently throughout Europe (Queloz *et al*., 2011).

As the *H. fraxineus* population has arrived recently in the UK and is expanding, the moderately high *G*_ST_ values observed between subpopulations reflect variation between the founder populations, not limited gene flow between them. When populations are not in mutation‐migration equilibrium, *G*_ST_ is not informative as a means of estimating gene flow. Even so, it can be concluded that at least two individuals, but very probably many more, initiated the subpopulation at each location because of the high genetic diversity in each subpopulation and because the pathogen is outcrossing, heterothallic and initiates infection by means of ascospores (Gross *et al*., 2014a).

It is not surprising that many individuals of *H. fraxineus* initiated ash dieback in planted woods, given the enormous scale of imports of ash stock into the UK, including from countries where the disease had established (Sansford, 2013). Infection of established woods such as LWD‐E and EPW‐E, where there was no history of recent planting, was most probably initiated by numerous *H. fraxineus* individuals through long‐distance dispersal of large numbers of ascospores from continental Europe. Although this has not been observed directly, the ascospores of *H. fraxineus* (mean 17 × 4.3 μm; Gross *et al*., 2012) are considerably smaller than conidia of *Blumeria graminis* (35 × 13 μm; Zhang *et al*., 2005). The latter can be transported for >550 km across the North Sea (Hermansen *et al*., 1978), so those of *H. fraxineus* may be wind‐dispersed even further. Although the majority of *H. fraxineus* ascospores are deposited close to their origin (Chandelier *et al*., 2014), airborne spores typically have a fat‐tailed distribution of dispersal distances in which a small proportion of spores can be transported very far from their source (Ferrandino, 1993). Hence, wind dispersal of *H. fraxineus* ascospores from disease‐affected locations in northwest continental Europe to eastern Great Britain (*c*. 75–500 km) is consistent with the biology of the fungus. The moderate differentiation of the subpopulations EPW‐E and LWD‐E suggests that they were initiated by separate ascospore populations from different locations on the continent. This points to the possibility of several episodes of ascospore infection of ash at diverse places in eastern Great Britain.

The various methods used in the present study to assess population diversity had different strengths and weaknesses. SNP markers were used because many potential markers could be found from genome sequence data generated from only a few isolates and also because they can be assessed rapidly. Moreover, SNPs typically have a small PCR product size, allowing poor‐quality DNA extracts to be used. This is advantageous because it has been difficult to obtain high molecular weight DNA from *H. fraxineus* (Downie, 2016). However, KASP‐based detection failed to identify SNP alleles from certain isolates even though the target sequence was present; the reason for this is unclear.

In tests of VC reactions to assess population diversity, there were occasional inconsistencies in scores on the different growth media. In particular, a few pairings were scored as compatible on ASA but incompatible on ALMA/MA, or vice versa. Such scores on each medium were repeatable, suggesting that environmental conditions, in this case the growing medium, may affect the expression of *vic* genes, at least *in vitro*, as has been previously reported for *Cryphonectria parasitica* (Cortesi *et al*., 1996). In a few instances the ‘triangle rule’ that if isolate A is compatible with B and B with C, then A and C should also be compatible was not observed. This may have been partly due to the tendency of *H. fraxineus* isolates to become unstable and to degenerate in culture, making it difficult to score some VC reactions accurately and to replicate tests with some pairs of isolates (Brasier & Webber, 2013; Fig. S2f). Moreover, just as the so‐called gene‐for‐gene system of host–parasite recognition can in fact involve complex interactions of several genes (Brown, 2002), it may be speculated that similar genetic complexity in the control of VC reactions could exist, especially in the case of poorly studied fungi such as *H. fraxineus*. This might give rise to an anomalous phenotype in a triangle of interactions.

The trading system within the European Union broadly assumes that planting stock is safe to move unless proved otherwise (Brasier, 2008). To support disease management within such a system, it is important to understand the risks and consequences of pathogen transmission. In turn, this requires knowledge of how pathogen populations are structured and the potential for further evolution, not only at the genome level, but also at the ecological and community levels. The present study has shown that vegetative compatibility and molecular data can be combined to generate a more complete picture of the population structure of *H. fraxineus*, as for *C. parasitica* (Liu *et al*., 1996). The high diversity of VC types in all UK subpopulations has implications for disease control, competition between genetic individuals and for natural selection (Brasier & Webber, 2013). Furthermore, it appears that the genetic diversity in the continental European populations of *H. fraxineus* has been transferred to the UK, both on imported nursery stock and as wind‐dispersed spores, with numerous infections establishing the pathogen at each location. It can be concluded that although trade in live plant material facilitated spread of *H. fraxineus* to the UK, the fungus would have become established in the UK, albeit more slowly, through wind dispersal of large numbers of ascospores from the continent even if imports of ash had been restricted from *c*. 2000 onwards. This indicates the need to establish and enforce stronger measures to prevent movement of diseased plant material not just between continental Europe and the UK, but into and within Europe (Brasier, 2008; Roy *et al*., 2014).

## Supporting information


**Figure S1** Reaction patterns of *Hymenoscyphus fraxineus* isolate pairings on ash sapwood agar (ASA) and ash leaf malt agar (ALMA) from six UK populations.

**Figure S2** Examples of gap reactions and aberrant growth in isolate pairings to test vegetative compatibility of *Hymenoscyphus fraxineus* collected in the UK in early 2014.

**Figure S3** Plot of the first three principal coordinates of single‐nucleotide polymorphism in *Hymenoscyphus fraxineus* isolates collected in Great Britain in spring 2014 and in continental Europe from 2008 to 2012.

**Table S1** Location and description of sampling sites for *Hymenoscyphus fraxineus* in England and Wales.

**Table S2** SNP markers found in the *Hymenoscyphus fraxineus* genome and the primer sets designed for each SNP marker location. The first 28 markers listed were used in the study.
