# Supplementary material for: Population structure of the ash dieback pathogen, Hymenoscyphus fraxineus, in relation to its mode of arrival in the UK
Source: Plant Pathol. 2017 Sep 26;67(2):255–64. doi: 10.1111/ppa.12762 (PMC5832303; doi:10.1111/ppa.12762)
Supplement: Supplementary file 1 — Figure S1 Reaction patterns of Hymenoscyphus fraxineus isolate pairings on ash sapwood agar (ASA) and ash leaf malt agar (ALMA) from six UK populations. [file PPA-67-255-s001.pdf]

EPW

060	CC												
061	C	CC											
066	G	g	CC										
068	G	G	g	CC									
070	G	G	G	G	CC								
071	G	G	G	G	G	CC							
072	G	C	G	G	g	G	CC						
073	G	G	g	G	g	G	G	CC					
076	g	C	G	g	g	-	C	g	CC				
077	G	G	G	G	G	G	g	G	g	CC			
079	g	G	G	G	G	-	G	G	G	G	CC		
082	G	g	G	G	G	G	G	G	C	G	G	Cc	
085	G	G	G	G	G	G	G	G	G	G	G	G	CC
ASA	060	061	066	068	070	071	072	073	076	077	079	082	085

060	CC												
061	C	CC											
066	G	g	CC										
068	G	G	G	CC									
070	G	g	G	G	CC								
071	G	g	G	G	G	CC							
072	g	C	G	G	G	-	CC						
073	G	g	g	G	G	G	G	CC					
076	g	C	g	g	g	-	C	g	CC				
077	g	G	G	G	G	G	g	G	G	CC			
079	g	g	G	G	g	-	G	G	G	g	CC		
082	G	g	G	G	G	G	g	G	G	g	G	cc	
085	G	G	G	G	G	G	g	G	G	G	G	G	CC
ALMA	060	061	066	068	070	071	072	073	076	077	079	082	085

ISC

003	Cc												
005	G	CC											
006	C	g	Cc										
009	g	G	g	Cc									
010	g	G	G	G	CC								
012	G	g	-	G	G	Cc							
013	g	-	g	g	G	g	CC						
014	G	G	G	G	G	G	G	CC					
015	G	G	G	G	G	G	g	-	CC				
016	G	g	G	g	g	G	G	C	g	CC			
019	C	g	g	g	-	C	g	G	G	g	CC		
020	g	g	g	g	G	g	G	G	G	G	-	C-	
021	g	G	g	g	C	g	G	G	G	G	g	g	Cc
ASA	003	005	006	009	010	012	013	014	015	016	019	020	021

003	Cc												
005	G	CC											
006	C	g	Cc										
009	g	G	g	Cc									
010	g	G	G	G	CC								
012	G	g	g	G	G	Cc							
013	g	-	g	G	G	g	CC						
014	G	G	G	G	G	G	G	CC					
015	g	G	g	G	G	G	g	-	CC				
016	G	g	G	g	g	G	G	C	g	CC			
019	C	g	g	g	-	G	g	G	G	g	CC		
020	-	g	-	-	g	g	G	G	G	G	-	C-	
021	g	G	g	g	C	g	G	G	G	G	g	-	c-
ALMA	003	005	006	009	010	012	013	014	015	016	019	020	021

LWD

044	CC									
045	G	CC								
049	G	G	cc							
053	G	G	G	CC						
054	G	G	G	G	CC					
065	G	G	G	G	G	CC				
066	G	G	G	C	G	G	CC			
067	G	G	g	G	G	G	G	CC		
069	G	G	G	G	G	G	G	G	cc	
070	G	G	G	G	G	G	g	G	G	CC
ASA	044	045	049	053	054	065	066	067	069	070

044	CC									
045	G	CC								
049	G	G	cc							
053	G	G	G	CC						
054	G	G	G	G	CC					
065	G	G	G	G	G	CC				
066	G	G	G	C	G	-	CC			
067	G	G	g	G	G	-	G	CC		
069	G	G	G	G	G	g	G	G	cc	
070	G	G	G	G	g	G	g	G	G	CC
ALMA	044	045	049	053	054	065	066	067	069	070

PTW

031	CC									
032	C	CC								
035a	G	g	CC							
036b	g	G	G	CC						
037b	G	G	G	G	CC					
039	C	G	C	G	g	CC				
041d	g	G	g	g	g	C	CC			
048	G	G	G	g	g	g	G	CC		
049c	g	C	C	G	g	g	G	G	Cc	
053	g	-	C	G	G	G	G	G	C	CC
ASA	031	032	035a	036b	037b	039	041d	048	049c	053

031	CC									
032	C	CC								
035a	G	G	CC							
036b	g	G	G	CC						
037b	G	G	G	G	CC					
039	C	G	C	G	g	CC				
041d	G	G	G	G	G	G	CC			
048	C	G	G	G	g	G	G	CC		
049c	g	C	C	G	G	g	G	G	CC	
053	G	g	C	G	G	g	G	g	C	CC
ALMA	031	032	035a	036b	037b	039	041d	048	049c	053

PND

002	CC									
003	G	CC								
006	G	g	CC							
007	G	G	G	Cc						
009	G	G	G	G	CC					
010	G	G	g	G	G	CC				
011	g	G	g	G	G	G	CC			
015	G	G	G	G	G	G	G	CC		
016	G	G	g	g	G	G	g	G	CC	
026	G	G	G	g	G	g	G	g	G	CC
ASA	002	003	006	007	009	010	011	015	016	026

002	CC									
003	G	CC								
006	g	g	CC							
007	g	G	G	Cc						
009	g	G	G	G	CC					
010	G	G	g	G	G	CC				
011	g	G	g	G	G	G	CC			
015	G	G	g	G	G	g	g	CC		
016	g	G	g	C	G	G	g	g	CC	
026	G	G	G	G	G	g	G	g	G	CC
ALMA	002	003	006	007	009	010	011	015	016	026

BWY

02L2	CC							
02L3	g	CC						
02L4	G	G	CC					
003	g	G	G	CC				
007	g	g	g	G	CC			
010	G	G	G	g	g	CC		
011	G	G	G	G	g	g	CC	
039	G	g	G	C	G	G	G	CC
ASA	02L2	02L3	02L4	003	007	010	011	039

02L2	CC							
02L3	g	CC						
02L4	G	G	CC					
003	g	G	G	CC				
007	g	g	g	G	cc			
010	G	G	G	g	C	CC		
011	G	G	G	G	g	g	CC	
039	G	g	G	C	G	G	G	CC
ALMA	02L2	02L3	02L4	003	007	010	011	039

Supplementary Figure 1. Reaction patterns of *Hymenoscyphus fraxineus* isolate pairings on ash sapwood agar (ASA) and ash leaf agar (ALMA) from six UK populations. C= Compatible, c= known compatible with unstructured, diffuse gaps, G= strong structured gap reaction, g= weaker structured gap reaction, - unreadable or failed reaction with no score.
