# Supplementary figures and images for: Population structure of the ash dieback pathogen, Hymenoscyphus fraxineus, in relation to its mode of arrival in the UK
Source: Plant Pathol. 2017 Sep 26;67(2):255–64. doi: 10.1111/ppa.12762 (PMC5832303; doi:10.1111/ppa.12762)

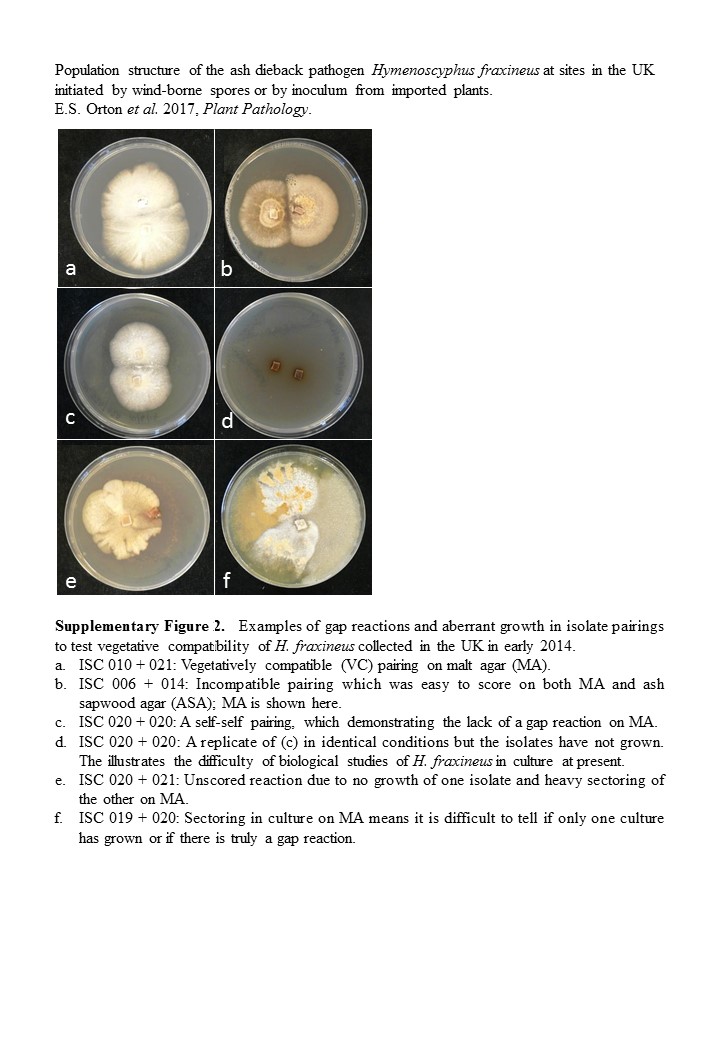

Supplement: Supplementary file 2 — Figure S2 Examples of gap reactions and aberrant growth in isolate pairings to test vegetative compatibility of Hymenoscyphus fraxineus collected in the UK in early 2014. [file PPA-67-255-s002.JPG]
