# Supplementary material for: Population structure of the ash dieback pathogen, Hymenoscyphus fraxineus, in relation to its mode of arrival in the UK
Source: Plant Pathol. 2017 Sep 26;67(2):255–64. doi: 10.1111/ppa.12762 (PMC5832303; doi:10.1111/ppa.12762)
Supplement: Supplementary file 3 — Figure S3 Plot of the first three principal coordinates of single‐nucleotide polymorphism in Hymenoscyphus fraxineus isolates collected in Great Britain in spring 2014 and in continental Europe from 2008 to 2012. [file PPA-67-255-s003.html]

Orton et al. 2017, Hymenoscyphus fraxineus population genetics.


# Population structure of *Hymenoscyphus fraxineus* in Great Britain and continental Europe

**Supplementary Figure 3.** 
Plot of the first three principal coordinates of single nucleotide polymorphism in
*Hymenoscyphus fraxineus* isolates collected in Great Britain in spring 2014 and in continental Europe from
2008 to 2012. Turn and zoom the interactive plot with a mouse or touchpad.  
Sites: L, LWD (eastern, established wood);
E, EPW (eastern, established);
P, PND (eastern, mixed);
B, BWY (western, planted);
I, ISC (western, planted);
T, PTW (Midlands, planted).
U, continental Europe.

---
