# Supplementary material for: Population structure of the ash dieback pathogen, Hymenoscyphus fraxineus, in relation to its mode of arrival in the UK
Source: Plant Pathol. 2017 Sep 26;67(2):255–64. doi: 10.1111/ppa.12762 (PMC5832303; doi:10.1111/ppa.12762)
Supplement: Supplementary file 4 — Table S1 Location and description of sampling sites for Hymenoscyphus fraxineus in England and Wales. [file PPA-67-255-s004.pdf]

Supplementary Table 1. Location and description of sampling sitess for *Hymenoscyphus fraxineus* in England and Wales.

Location code	Location	Site description	Sample type	Sampling date	Sampled / isolated by	Number of isolates	Isolate codes
**Isolated planting-associated outbreaks**							
BWY *Planted*	Tiverton Devon	Planted in 1996 with some replacement blocks of mixed broadleaf tree species.	Branches and shoots 2-4 cm diameter on ash 10-15 cm diameter at breast height (dbh).	November 2013	CB , KK	3	BWY 002 L1-L3
				April 2014	CB, KK	40	BWY 003-042
ISC *Planted*	Whitland Carmarthenshire	Planted in 2006 on grazing site. Close to older ash planted in 1990s, also becoming symptomatic in the canopy.	Ash shoots/branches, mostly 1-2 cm dbh.	March 2014	OT, KK	24	ISC 001-024
PTW *Planted*	Swadlingcote Derbyshire	Two 2008 planting blocks on ex mine site with trees planted in 1997 also present.	Ash stems and branches, 1.5-3 cm dbh.	December 2013	BJ , KK	10	PTW 001-010
				April 2014	CB , KK	21	PTW 0030-050
**East coast 'widespread infection' zone**							
EPW *Established*	Canterbury Kent	Mature mixed broadleaf and conifer plantation with natural ash stands and patches of ash regeneration.	Stems and branches on regeneration saplings mostly 1-5 cm dbh close to infected stands of mature ash.	March 2014	EO, CB	26	EPW 060-085
LWD *Established*	Wymondham Norfolk	Ancient ash and hazel woodland, with ash coppice and mature standards.	Coppice ash stems 2-5 cm dbh.	March 2014	EO ,JB	33	LWD 042-070
PND *Mixed*	Saxmundham Suffolk	Mixed broadleaf woodland with some planted areas ca 1993-2002.	Stems and branches from natural regeneration saplings, mostly 1-5 cm dbh.	March 2014	EO, JB, CB	26	PND 001-026

BJ, Ben Jones (Forestry Commission); CB, Clive Brasier; EO, Elizabeth Orton; JB, James Brown; KK, Kevin King (Forest Research); OT, Owen Thurgate (FC)
