# Supplementary material for: Population structure of the ash dieback pathogen, Hymenoscyphus fraxineus, in relation to its mode of arrival in the UK
Source: Plant Pathol. 2017 Sep 26;67(2):255–64. doi: 10.1111/ppa.12762 (PMC5832303; doi:10.1111/ppa.12762)
Supplement: Supplementary file 5 — Table S2 SNP markers found in the Hymenoscyphus fraxineus genome and the primer sets designed for each SNP marker location. The first 28 markers listed were used in the study. [file PPA-67-255-s005.pdf]

Marker location	SNP	Allele 1 primer(A)	Allele 2 primer (B)	Common primer	Orientation	Product size
[1]Cf746836_TGAC_s1v1_scaffold_10:11517-11517	C183T	tcgaagagcaataaatctttgtgC	tcgaagagcaataaatctttgtgT	tgctgagggtatgcgaatca	forward	75
[1]Cf746836_TGAC_s1v1_scaffold_1002:17412-17412	G160A	atcgattcgtcttgcgccG	atcgattcgtcttgcgccA	gagcggaaacagctctacga	forward	87
[1]Cf746836_TGAC_s1v1_scaffold_1129:18698-18698	G97A	ttaccacgatcgcttgtatcG	ttaccacgatcgcttgtatcA	accaaaccaacccaggtgaa	forward	67
[1]Cf746836_TGAC_s1v1_scaffold_1163:15555-15555	A183G	cgtattagccttgattggtagtgtA	cgtattagccttgattggtagtgtG	agacgttgccgcggatatac	forward	55
[1]Cf746836_TGAC_s1v1_scaffold_1206:21784-21784	C61T	gacacttcaattgcagggcC	gacacttcaattgcagggcT	tgctacacaccagatgcacg	forward	50
[1]Cf746836_TGAC_s1v1_scaffold_1207:16186-16186	C106A	cgaagcctggagattcgcC	cgaagcctggagattcgcA	cccactgtagccgttcaact	forward	62
[1]Cf746836_TGAC_s1v1_scaffold_1208:18637-18637	A127G	acctatctttgccatcgttggA	acctatctttgccatcgttggG	cgcggatcattggggttagt	forward	93
[1]Cf746836_TGAC_s1v1_scaffold_1214:14499-14499	C257T	agacgtttatcgaagcatctacC	agacgtttatcgaagcatctacT	acccttcgttctgatgagcg	forward	50
[1]Cf746836_TGAC_s1v1_scaffold_1222:16284-16284	G242C	ctcctacgcaaatatcaccgaaaG	ctcctacgcaaatatcaccgaaaC	tttctctacgggctcctcca	forward	50
[1]Cf746836_TGAC_s1v1_scaffold_1224:31390-31390	C96A	ccaatgctgtcgacaatggaaC	ccaatgctgtcgacaatggaaA	ggaggttgatacgtcgattcgt	forward	56
[1]Cf746836_TGAC_s1v1_scaffold_1314:26104-26104	A75G	ccatatagacgacaaattcagccT	ccatatagacgacaaattcagccC	atgcgcacctgtaccacaaa	reverse	58
[1]Cf746836_TGAC_s1v1_scaffold_1654:48638-48638	C197A	ccgagtgggaatgaatcagataaC	ccgagtgggaatgaatcagataaA	gtctcaagtcagcggcctaa	forward	56
[1]Cf746836_TGAC_s1v1_scaffold_190:7813-7813	A181G	gccgaaagtgttgatctaccaaaA	gccgaaagtgttgatctaccaaaG	gcgaggcattgacttgtagc	forward	55
[1]Cf746836_TGAC_s1v1_scaffold_230:7222-7222	T256C	tcgaaacggaactcccaataT	tcgaaacggaactcccaataC	tcaacattacccccggcttc	forward	50
[1]Cf746836_TGAC_s1v1_scaffold_260:3927-3927	A174G	gtggcagtctatcctcacagA	gtggcagtctatcctcacagG	tccccgctctaccgaataga	forward	87
[1]Cf746836_TGAC_s1v1_scaffold_266:4074-4074	C173T	tcaaacgacttacaagcaacagC	tcaaacgacttacaagcaacagT	gggcctcgatacaaccacaa	forward	52
[1]Cf746836_TGAC_s1v1_scaffold_370:2537-2537	C131T	gcctgcttcttccccttagC	gcctgcttcttccccttagT	ggctaatcgtgctcctgcta	forward	57
[1]Cf746836_TGAC_s1v1_scaffold_435:4691-4691	G131A	gaggagacgagcgagccG	gaggagacgagcgagccA	ccacctcccaaagtactccc	forward	61
[1]Cf746836_TGAC_s1v1_scaffold_503:8173-8173	T131C	tctcaatccattggcttggT	tctcaatccattggcttggC	attgcctcaaacgactgctg	forward	69
[1]Cf746836_TGAC_s1v1_scaffold_570:12487-12487	C131T	cagaaggctacaacgtggaC	cagaaggctacaacgtggaT	gcgttcatatcggccgtttg	forward	77
[1]Cf746836_TGAC_s1v1_scaffold_641:18581-18581	T113C	acgtctggtaaatgcatcgaaT	acgtctggtaaatgcatcgaaC	ggaggggaggcaatattccg	forward	54
[1]Cf746836_TGAC_s1v1_scaffold_721:14083-14083	T216C	ccgaagttacactgcccatatT	ccgaagttacactgcccatatC	tgggaaaggcttggaaggag	forward	54
[1]Cf746836_TGAC_s1v1_scaffold_910:18473-18473	C145T	ccctttggctcactggatcC	ccctttggctcactggatcT	tactttcctgactccgcagt	forward	50
[1]Cf746836_TGAC_s1v1_scaffold_926:10136-10136	C183A	tcgaggcagaaggatattacgaC	tcgaggcagaaggatattacgaA	gccttcatgcgaatatcggc	forward	73
[1]Cf746836_TGAC_s1v1_scaffold_943:42592-42592	T131C	tttttgttctttggtgccactaT	tttttgttctttggtgccactaC	ggcgtcccctttgtatagca	forward	59
[1]Cf746836_TGAC_s1v1_scaffold_945:18575-18575	C183A	agcatcgactcacacagaattaaC	agcatcgactcacacagaattaaA	atatgttcccgatcggcctg	forward	55
[1]Cf746836_TGAC_s1v1_scaffold_954:35750-35750	G184A	tcggcctttagcacttccaG	tcggcctttagcacttccaA	ttacaatcccggcgacactg	forward	102
[1]Cf746836_TGAC_s1v1_scaffold_960:13477-13477	T132C	agcaacttcggaagagtggtT	agcaacttcggaagagtggtC	taagccaccggatcgatgag	forward	50
Cf746836_TGAC_s1v1_scaffold_10:3268-3268	A92G	gggacacaaccacaggagaA	gggacacaaccacaggagaG	atccggccatacaacagcat	forward	50
Cf746836_TGAC_s1v1_scaffold_1000:32711-32711	C131T	gttctcaaattggatagggagtatG	gttctcaaattggatagggagtatA	actcttcctcgacacccgt	reverse	94
Cf746836_TGAC_s1v1_scaffold_1005:8542-8542	A167T	ggatgctttgatatgtgtatacgaT	ggatgctttgatatgtgtatacgaA	cccggaatcacctcacaaca	reverse	69

Marker location	SNP	Allele 1 primer(A)	Allele 2 primer (B)	Common primer	Orientation	Product size
Cf746836_TGAC_s1v1_scaffold_1005:9139-9139	T95G	tcgaaacatatgaagcgactcT	tcgaaacatatgaagcgactcG	tcgcacccaggcgaattg	forward	73
Cf746836_TGAC_s1v1_scaffold_1011:10313-10313	G184A	tttctgaggcttttccaactttC	tttctgaggcttttccaactttT	attctccttggcccaatccg	reverse	52
Cf746836_TGAC_s1v1_scaffold_1015:13833-13833	G162A	tttctgaggcttttccaactttC	tttctgaggcttttccaactttT	attctccttggcccaatccg	reverse	52
Cf746836_TGAC_s1v1_scaffold_107:6372-6372	A226C	agacggcgatgggagatcA	agacggcgatgggagatcC	tccagcaaccagtcgttctc	forward	110
Cf746836_TGAC_s1v1_scaffold_1119:10948-10948	T131A	acgacgggccatatcttgaT	acgacgggccatatcttgaA	tgactaccttctgctcccca	forward	83
Cf746836_TGAC_s1v1_scaffold_1127:10226-10226	C149T	ggagagagtaaggggttggaC	ggagagagtaaggggttggaT	gtcgtcgtcaagctcctcat	forward	70
Cf746836_TGAC_s1v1_scaffold_1135:21042-21042	G177A	cttccaagttcgagtagcaacG	cttccaagttcgagtagcaacA	tgtggacttcttgcaatgacc	forward	50
Cf746836_TGAC_s1v1_scaffold_1153:17716-17716	C184T	tgataagtatgcggatgagaaaagG	tgataagtatgcggatgagaaaagA	gaccctactccaatcccgtt	reverse	81
Cf746836_TGAC_s1v1_scaffold_1154:19073-19073	A81T	ccgtccatgcgctccatttT	ccgtccatgcgctccatttA	ggacacagagcccttgtcat	reverse	75
Cf746836_TGAC_s1v1_scaffold_1165:20102-20102	G190A	agctaacaagtgcatcgcaatG	agctaacaagtgcatcgcaatA	tggccgatacagaacaacga	forward	68
Cf746836_TGAC_s1v1_scaffold_1173:10486-10486	C90A	ggagtttgatgactttactctcgtG	ggagtttgatgactttactctcgtT	tccgtcaccaagattgtcca	reverse	106
Cf746836_TGAC_s1v1_scaffold_1176:13698-13698	A133G	attccccaccaagtctcgaT	attccccaccaagtctcgaC	tggatacatgcaactggcgt	reverse	58
Cf746836_TGAC_s1v1_scaffold_1177:36419-36419	T255C	catccgaccgcttgacagaT	catccgaccgcttgacagaC	ttggaaacggacgactcctg	forward	50
Cf746836_TGAC_s1v1_scaffold_1178:12140-12140	G185A	gttgccattgttctacataggtcC	gttgccattgttctacataggtcT	taggagatcgaccctggctc	reverse	73
Cf746836_TGAC_s1v1_scaffold_1188:15873-15873	G185A	cggtgtgcatgatcttcagaaG	cggtgtgcatgatcttcagaaA	tcttgtcgcggaggttttct	forward	50
Cf746836_TGAC_s1v1_scaffold_1191:8950-8950	T82C	ccccaaactgtctccatccT	ccccaaactgtctccatccC	cctcagtgcatcggaccttt	forward	50
Cf746836_TGAC_s1v1_scaffold_1192:52144-52144	C182T	cccacagttcagtcatgcgG	cccacagttcagtcatgcgA	tcacgcggggagacaattaa	reverse	69
Cf746836_TGAC_s1v1_scaffold_1194:14893-14893	G255A	tctcgctggattatgggaagataG	tctcgctggattatgggaagataA	cgtgtttccaggtgaggtca	forward	53
Cf746836_TGAC_s1v1_scaffold_1195:40301-40301	C180T	tcaactaccttgtagacgtgtatC	tcaactaccttgtagacgtgtatT	atcctgccgcgaagaaactt	forward	87
Cf746836_TGAC_s1v1_scaffold_1197:11925-11925	T183C	acctaacatacagttgaggagcaT	acctaacatacagttgaggagcaC	tgaggagaacgttcgcacat	forward	53
Cf746836_TGAC_s1v1_scaffold_1200:67205-67205	T188C	tgaatccagctatcgacctcA	tgaatccagctatcgacctcG	gctgagaagtgggaaggacc	reverse	50
Cf746836_TGAC_s1v1_scaffold_1202:34718-34718	A148T	cctcgatgcctagtcttttagctA	cctcgatgcctagtcttttagctT	ttcgtcccccgattctctct	forward	54
Cf746836_TGAC_s1v1_scaffold_1203:31603-31603	A211G	tgattctcgagtctttttgatggA	tgattctcgagtctttttgatggG	ccatggaatttgtggagtctatgc	forward	72
Cf746836_TGAC_s1v1_scaffold_1228:9887-9887	C78T	atagctttatcttaccgcctaaaaG	atagctttatcttaccgcctaaaaA	agtctagagcttagtagggtgaa	reverse	61
Cf746836_TGAC_s1v1_scaffold_1234:36065-36065	T129G	aatctgtctcccaacggtcT	aatctgtctcccaacggtcG	cgacgtggggatgaaaatgc	forward	52
Cf746836_TGAC_s1v1_scaffold_1235:24111-24111	G106A	ccaactccattcgcttctcaC	ccaactccattcgcttctcaT	cggagtggtttgttgggttg	reverse	61
Cf746836_TGAC_s1v1_scaffold_1237:18853-18853	T257C	cgatattctcggtatccccagT	cgatattctcggtatccccagC	aaaggagaaaggagggcagg	forward	50
Cf746836_TGAC_s1v1_scaffold_1238:11063-11063	C158T	tcacgtgtcttgctatgaactC	tcacgtgtcttgctatgaactT	cttcctttcggaccgttcgt	forward	57
Cf746836_TGAC_s1v1_scaffold_1240:16580-16580	G209A	tcaacatgaatctagtgtctagcC	tcaacatgaatctagtgtctagcT	gaaggtaccaagctgagagc	reverse	79
Cf746836_TGAC_s1v1_scaffold_1249:12785-12785	G133C	accttatccccatcattcctttctC	accttatccccatcattcctttctG	atcgagggtgaaggtgagga	reverse	50
Cf746836_TGAC_s1v1_scaffold_1259:17360-17360	A112G	tggataaacaaaccttggccA	tggataaacaaaccttggccG	acctgcttggatgttctatgtt	forward	55
Cf746836_TGAC_s1v1_scaffold_130:5372-5372	G178A	ttggaggacgagtgcagtaC	ttggaggacgagtgcagtaT	cctctctccctcatcctgca	reverse	118
Cf746836_TGAC_s1v1_scaffold_136:10532-10532	C183G	ccacttcccgtgcatcgtC	ccacttcccgtgcatcgtG	ttttcagggttaccgcggg	forward	88
Cf746836_TGAC_s1v1_scaffold_1475:21355-21355	G77C	acacataacccgaccctcaC	acacataacccgaccctcaG	gctggggtttgttttgctca	reverse	61
Cf746836_TGAC_s1v1_scaffold_1489:19529-19529	G278A	tgtttcccgcctactatcagaG	tgtttcccgcctactatcagaA	tcgcctagagagcctctact	forward	57

Marker location	SNP	Allele 1 primer(A)	Allele 2 primer (B)	Common primer	Orientation	Product size
Cf746836_TGAC_s1v1_scaffold_160:1888-1888	A163G	cgtagacagtttgaatctcacgA	cgtagacagtttgaatctcacgG	gatgttgctgcgctagagga	forward	66
Cf746836_TGAC_s1v1_scaffold_19:9684-9684	C121T	actcattccatcttctatgtcttcC	actcattccatcttctatgtcttcT	cttcttgatcgagtgcgcgt	forward	50
Cf746836_TGAC_s1v1_scaffold_20:26048-26048	T213C	ctccaaagtcttgacagcaaataT	ctccaaagtcttgacagcaaataC	tcgactgacttggccttgtc	forward	53
Cf746836_TGAC_s1v1_scaffold_250:5824-5824	A211T	tggactattcgggatatgcaaagA	tggactattcgggatatgcaaagT	ccctcatccccaaaatccaca	forward	95
Cf746836_TGAC_s1v1_scaffold_290:1783-1783	C132T	gcgtatatgtcggctggtC	gcgtatatgtcggctggtT	ggcctctctctcttttcgca	forward	64
Cf746836_TGAC_s1v1_scaffold_31:14199-14199	C183A	gctcaacggctttccaccC	gctcaacggctttccaccA	agcaacctgcctactttggt	forward	68
Cf746836_TGAC_s1v1_scaffold_320:8266-8266	C131T	aactccacgatatattgccatcC	aactccacgatatattgccatcT	cctcgcgtcacaaacctaac	forward	108
Cf746836_TGAC_s1v1_scaffold_360:3688-3688	G202A	gggaccgcggtttgtcttC	gggaccgcggtttgtcttT	gagcttcacgtttgatgggc	reverse	65
Cf746836_TGAC_s1v1_scaffold_373:5593-5593	C142T	aagattggccaccagcgaG	aagattggccaccagcgaA	ccgaggatgccaagtctcta	reverse	65
Cf746836_TGAC_s1v1_scaffold_38:12528-12528	A184C	ccaaaaacactggtacgtcgA	ccaaaaacactggtacgtcgC	cgagctatgcgtccaaacca	forward	104
Cf746836_TGAC_s1v1_scaffold_390:3663-3663	A75G	ggagagccgagtagaggtgT	ggagagccgagtagaggtgC	gacggaattcaggagggaca	reverse	64
Cf746836_TGAC_s1v1_scaffold_395:16425-16425	T257A	agctttgctgagaacgcctA	agctttgctgagaacgcctT	agacttcatggaagctccca	reverse	52
Cf746836_TGAC_s1v1_scaffold_405:12701-12701	C251T	actgccttgaacaagtcagaataG	actgccttgaacaagtcagaataA	aatggtggagagccttcagg	reverse	52
Cf746836_TGAC_s1v1_scaffold_411:12709-12709	T84G	cggggtgtgggatcgagaA	cggggtgtgggatcgagaC	gcctttcgtaaatccatgtggg	reverse	51
Cf746836_TGAC_s1v1_scaffold_430:16527-16527	G127A	tctggatcgtagacaaatacaacG	tctggatcgtagacaaatacaacA	tggacgcgatcgaaaaggtt	forward	61
Cf746836_TGAC_s1v1_scaffold_440:6854-6854	G131A	ggagccacaccattgaagcC	ggagccacaccattgaagcT	tggggctacatcaacactcg	reverse	61
Cf746836_TGAC_s1v1_scaffold_461:15064-15064	A183G	aagtgtaggaattaaagatgcatcT	aagtgtaggaattaaagatgcatcC	cagcatgtggaacgttgcat	reverse	101
Cf746836_TGAC_s1v1_scaffold_502:8343-8343	G109T	gtccttttctatgttggcatactcC	gtccttttctatgttggcatactcA	ggtatcggcaatccaggcaa	reverse	58
Cf746836_TGAC_s1v1_scaffold_522:8890-8890	T107C	ctgcacctttggccaatgaT	ctgcacctttggccaatgaC	aaatgctgccggtaccagac	forward	50
Cf746836_TGAC_s1v1_scaffold_537:3328-3328	A183G	cccccatagatcaacacctgA	cccccatagatcaacacctgG	catctcgaggagctacagtgt	forward	80
Cf746836_TGAC_s1v1_scaffold_545:37645-37645	A183T	agcacggatcaaatggataaatgT	agcacggatcaaatggataaatgA	atcgagggtatatagagcatttgat	reverse	76
Cf746836_TGAC_s1v1_scaffold_591:13027-13027	A131G	catctctacccatgtcgaacatT	catctctacccatgtcgaacatC	gtggcggggtcttcaatgta	reverse	69
Cf746836_TGAC_s1v1_scaffold_610:18880-18880	G114A	gtctgtgaagacattctccaaattC	gtctgtgaagacattctccaaattT	aggaaagattcttgaatccccga	reverse	104
Cf746836_TGAC_s1v1_scaffold_630:22098-22098	A141G	caacctgggcagtctctagT	caacctgggcagtctctagC	tgcggaaatagtcgtgcact	reverse	64
Cf746836_TGAC_s1v1_scaffold_692:4013-4013	G148A	ccccaccacaccactttcG	ccccaccacaccactttcA	tctggttcaagagtcgtggag	forward	50
Cf746836_TGAC_s1v1_scaffold_70:14807-14807	C183T	ggcgtctccatgtagaagcG	ggcgtctccatgtagaagcA	gatgaccgtcgtgtccctac	reverse	52
Cf746836_TGAC_s1v1_scaffold_701:12513-12513	G256C	tgtgactcctagtccccgG	tgtgactcctagtccccgC	ctgtgagaacctgcatcggg	forward	59
Cf746836_TGAC_s1v1_scaffold_703:6096-6096	C81T	tgagtgcaacgaatgattggG	tgagtgcaacgaatgattggA	ctcccaactaacagccaccc	reverse	59
Cf746836_TGAC_s1v1_scaffold_715:3029-3029	C130A	acaccgacgatcaggagaatC	acaccgacgatcaggagaatG	acttgaagattgccaccccc	forward	75
Cf746836_TGAC_s1v1_scaffold_719:5776-5872	C107A	tgcaagacagtccatggtcG	tgcaagacagtccatggtcT	ttccttctgggatgggtgga	reverse	50
Cf746836_TGAC_s1v1_scaffold_725:17813-17813	C58T	gaagaggttgcagttggctG	gaagaggttgcagttggctA	tgccccagtatccccagatt	reverse	50
Cf746836_TGAC_s1v1_scaffold_745:7897-7897	G115A	acgtaatatcaacggcactacG	acgtaatatcaacggcactacA	ggctatggttgggggaacag	forward	72
Cf746836_TGAC_s1v1_scaffold_751:17953-17953	G109A	tcccgatttagaagagtacttgttC	tcccgatttagaagagtacttgttT	cgcggatatatccttaatagagagg	reverse	67
Cf746836_TGAC_s1v1_scaffold_757:14001-14001	T65C	accttgggtgttcctcatttT	accttgggtgttcctcatttC	ccaagactgatccaccttgca	forward	94
Cf746836_TGAC_s1v1_scaffold_763:35469-35469	G175A	tcgcgcccctagcctagC	tcgcgcccctagcctagT	actttcgagagctaggtagaattaa	reverse	52

Marker location	SNP	Allele 1 primer(A)	Allele 2 primer (B)	Common primer	Orientation	Product size
Cf746836_TGAC_s1v1_scaffold_767:14435-14435	G132A	tcgctcgttcgacctgaC	tcgctcgttcgacctgaT	gcgacgtgtatccttgggtt	reverse	53
Cf746836_TGAC_s1v1_scaffold_782:14949-14949	C87G	gccgacactgatagcgcC	gccgacactgatagcgcG	ggccttttcaaattgaccaagc	forward	69
Cf746836_TGAC_s1v1_scaffold_790:20054-20054	G151A	tggcgattggtactctacagaC	tggcgattggtactctacagaT	atgccttgtactgcttccgt	reverse	50
Cf746836_TGAC_s1v1_scaffold_803:17894-17894	A107C	ggtggcgaggaagagctgT	ggtggcgaggaagagctgG	agccaattcccaaaatgcgc	reverse	50
Cf746836_TGAC_s1v1_scaffold_811:20379-20379	T141C	cgttctattatgcactagccgaA	cgttctattatgcactagccgaG	ggtttgctgcggattggaag	reverse	50
Cf746836_TGAC_s1v1_scaffold_817:16085-16085	T119C	tcttccattttcgacgagcataT	tcttccattttcgacgagcataC	gtgtggaaggaaattgccgg	forward	59
Cf746836_TGAC_s1v1_scaffold_821:7573-7573	G184A	cgcatcactttatcccagtattatC	cgcatcactttatcccagtattatT	cgtttcgatggcggaattgt	reverse	79
Cf746836_TGAC_s1v1_scaffold_845:7781-7781	G132A	attgggttgcttcctcggaC	attgggttgcttcctcggaT	aaccttgcctgtcgattgct	reverse	51
Cf746836_TGAC_s1v1_scaffold_860:9331-9331	A183G	actcgtgatgcgcaattacA	actcgtgatgcgcaattacG	gttcgagaccctgtgactgg	forward	79
Cf746836_TGAC_s1v1_scaffold_865:15100-15100	C183T	tctcgttatacagccagcgtC	tctcgttatacagccagcgtT	cgagatgaagtccagaccttga	forward	70
Cf746836_TGAC_s1v1_scaffold_880:6319-6319	T135G	acaagtaaacagactcccccatT	acaagtaaacagactcccccatG	attcacgtgtgtggtgcaga	forward	59
Cf746836_TGAC_s1v1_scaffold_890:49790-49790	C135T	agatcaaggtcgaagaggtgG	agatcaaggtcgaagaggtgA	gtgacgaccttctcctcgac	reverse	79
Cf746836_TGAC_s1v1_scaffold_915:18417-18417	T183A	ttctgaagaagtgtacagaaggaT	ttctgaagaagtgtacagaaggaA	tgcgcctcaacatggtaaga	forward	51
Cf746836_TGAC_s1v1_scaffold_935:9476-9476	G152A	tgactacgatttgcccccaG	tgactacgatttgcccccaA	tgctgttttcctccactgct	forward	56
Cf746836_TGAC_s1v1_scaffold_967:37083-37083	T184C	cctatttaaccgttccgttaccttA	cctatttaaccgttccgttaccttG	aatcgggctaagcgtcgg	reverse	76
Cf746836_TGAC_s1v1_scaffold_970:12597-12597	G132A	taggaatgacggctgtgtcC	taggaatgacggctgtgtcT	tggaaactactcctcgaccct	reverse	78
Cf746836_TGAC_s1v1_scaffold_980:47596-47596	G184T	tgatggtgatgaatgaccaaacG	tgatggtgatgaatgaccaaacT	caccgagaatctcagcgact	forward	57
Cf746836_TGAC_s1v1_scaffold_986:25597-25597	T116C	acacattcgctctcagcagT	acacattcgctctcagcagC	tagagtccccgcagcagat	forward	80

[1] Markers used for population study.
